# Uncommon adrenal rest tumors and massive adrenal enlargement in adult with congenital adrenal hyperplasia mimicking metastasis from pleomorphic sarcoma

**DOI:** 10.1186/s12902-024-01635-z

**Published:** 2024-07-08

**Authors:** Pierluigi Mazzeo, Irene Tizianel, Francesca Galuppini, Marta Sbaraglia, Mattia Barbot

**Affiliations:** 1 Department of Medicine DIMED, University of Padua, Padua, Italy; 2 Endocrinology Unit, Department of Medicine DIMED, University-Hospital of Padua, Via Ospedale Civile, Padua, 105 - 35128 Italy; 3 Pathology Unit, University-Hospital of Padua, Padua, Italy

**Keywords:** Congenital adrenal hyperplasia, 21-hydroxylase deficiency, Testicular adrenal rest tumors, Ectopic adrenal rest tumor, Case report

## Abstract

**Background:**

Congenital adrenal hyperplasia (CAH) encompassed a bunch of autosomal recessive disorders characterized by impaired cortisol levels due to an enzymatic deficiency in steroid synthesis. In adult male patients with CAH, a frequent complication related to poor disease control is the development of ectopic adrenocortical tissue in the testes, named testicular adrenal rest tumors (TART). Conversely, ovarian adrenal rest tumors (OART) in females are extremely rare and adrenal rests in sites other than gonads are so uncommon to have been described only few times in literature.

**Case presentation:**

We report a case of a male patient with untreated CAH and oncologic history of pleomorphic sarcoma who presented with massive bilateral adrenal enlargement and adrenal rest tumors in peri-lumbar and peri-cecal sites, which mimicked metastasis from sarcoma.

**Conclusions:**

The development of massive adrenal enlargement and ectopic adrenal rest tumors in sites other than gonads, even if very uncommon, should be suspected in patients with CAH and prolonged periods of undertreatment.

## Background

Congenital adrenal hyperplasia (CAH) is a group of autosomal recessive disorders characterized by impaired cortisol production due to an enzymatic deficiency of steroid synthesis. The 21-hydroxylase deficiency (21-OHD) is by far the most frequent cause of CAH accounting for more than 90% of all cases [1].

Based on residual enzymatic activity, it is distinguished in classic and non-classic CAH; the former is a severe condition associated with high perinatal mortality before the introduction of newborn screening, especially in its salt-wasting form, whereas the latter is a mild impairment with variable degrees of postnatal androgen excess that can range from precocious puberty to a complete asymptomatic condition [1, 2].

Glucocorticoids are the mainstay of CAH treatment with the double aim of preventing adrenal insufficiency and suppressing androgen excess through a direct negative feedback to the pituitary adrenocorticotropic hormone (ACTH) secretion [1, 3]. However, maintaining an adequate balance between the need for androgen suppression while avoiding glucocorticoid overtreatment is a delicate task, since both conditions are burdened by increased morbidity and mortality [1].

Development of TART is quite a common complication in adult male patients with CAH and it is mainly related to poor disease control [4]. It has been hypothesized that TART derives from the proliferation of adrenal cortex remnants in the testicular area; however, recent studies speculate that they originate from a population of adrenal-like pluripotent stem cells, already present at the gonadal site during embryogenesis, that undergo adrenal differentiation; these cells proliferate in response to high ACTH levels with consequent hyperplastic growth [2]. This hypothesis is supported by the presence of TART also in other conditions with elevated plasma ACTH concentrations such as Nelson’s syndrome [5]; moreover, the expression of adrenal-specific steroidogenic enzymes receptors as well as ACTH and angiotensin II receptors was demonstrated in TART-derived tissue [6].

Apart from CAH undertreatment, other factors seem to be involved in TART development, as some patients with TART are not undertreated and show serum levels of ACTH within the normal range [7].

First, the presence of luteinizing hormone (LH) receptor gene expression in TART and the increased prevalence of TART in adolescence, suggested that the pubertal rise of LH may also play a role in developing or promoting the growth of these adrenal rest cells [8].

Moreover, a modulating role of inflammatory cells has also been suggested, as lymphocytic aggregates were found in adrenal rest tumors, and transcriptional data revealed an increase expression of pro-inflammatory cytokines [9].

However, TART have a prevalence ranging from 0 to 94% of male patients with CAH, depending on age, CAH genotype and disease control [2, 7].

Although adrenal rest tumors are quite common in adult male patients, ovarian adrenal rest tumors (OART) in females are extremely rare and, above all, adrenal rests in sites other than gonads are so uncommon to have been described few times in the literature [10, 11].

Here we described a case of a patient with CAH due to 21-hydroxylase deficiency that developed chronic complications due to almost thirty years of pharmacological treatment interruption; the results of an uncontrolled disease were the presence of massive bilateral adrenal enlargement, TART and, notably, peri-lumbar and peri-cecal adrenal rest tumors, an extremely unusual complication.

## Case presentation

A 45-years old male with CAH was referred to our Hospital for endocrine assessment after finding massive bilateral adrenal enlargement.

The patient had a history of pleomorphic sarcoma, diagnosed in March 2021, when he sought medical evaluation for a lesion on the left upper arm that had increased in size in the previous months. First of all, he performed an ultrasound evaluation documenting a vascularized mass of 5 cm; later, a magnetic resonance (MR) imaging confirmed the presence of a solid mass of 5.3 × 3.1 cm between the triceps and biceps without a cleavage plane, that reached the cortical bone. Due to its suspicious features, the lesion was surgically removed and the histological finding was consistent with undifferentiated pleomorphic sarcoma of intermediate grade (G2).

Therefore, he went through a second surgery for radicalization in November 2021 and adjuvant local radiotherapy.

**Fig. 1:**
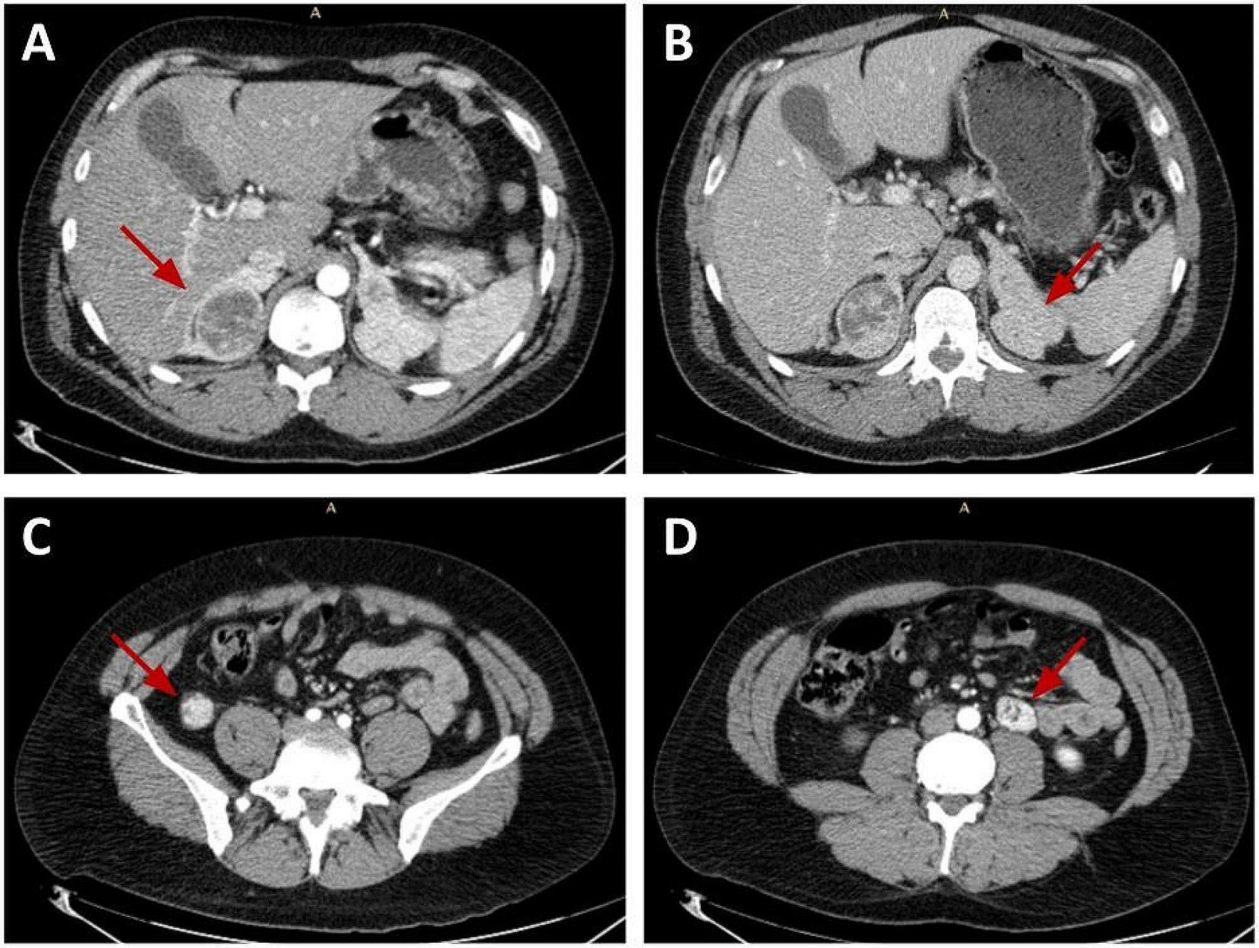
Contrast enhanced CT scan: (**A**) Right adrenal gland lesion of 5 cm with a central colliquation area; (**B**) major left adrenal gland lesions of 4,2 cm; (**C**) paralumbar right lesion of 2.6 cm; (**D**) lesion in left peri-cecal fat of 3.1 cm

Notably, during tumor staging, the CT scan showed bilateral adrenal hyperplasia with the presence of two hyperdense round formations of 4.2 and 2.6 cm on the left adrenal gland and one lesion of 5 cm with a central colliquative area on the right gland (Fig. 1). Furthermore, other two lesions with the same densitometric characteristics were detected on paralumbar right side and within peri-cecal fat, that measured 2.6 cm and 3.1 cm respectively (Fig. 1). Thus, the patient was submitted to a biopsy of the peri-cecal lesion that was consistent with adrenal tissue.

Due to this finding, it was referred to our Unit for endocrine assessment.

### Investigation

While reviewing the patient’s medical history, we found that he was diagnosed at birth with CAH, but at the time of our first evaluation, he was not taking any medication.

Indeed, he was treated with glucocorticoid and mineralocorticoid therapy up to the age of 16, when he autonomously decided to discontinue pharmacological treatment and medical follow-up.

Laboratory findings showed the presence of uncontrolled disease, with increased ACTH (123 ng/L, normal values 4.7–48.8 ng/L) and 17-hydroxyprogesterone levels (2600 nmol/L, normal values 1.52–6.36 nmol/L); serum electrolytes were normal (sodium 137 nmol/l, potassium 4.1 nmol/L), while morning serum cortisol was reduced (137 nmol/L).

Genetic analysis revealed the presence of *CYP21A2* mutation, in homozygosis (variant C.428T > C_p.Leu148Pro), consistent with the diagnosis of simple virilizing (SV) CAH due to 21-OHD.

Ultrasound testicular evaluation detected a slight reduction in testicles’ dimensions, with diffuse inhomogeneous areas all over the testicular parenchyma compatible with diffuse TART.

**Fig. 2:**
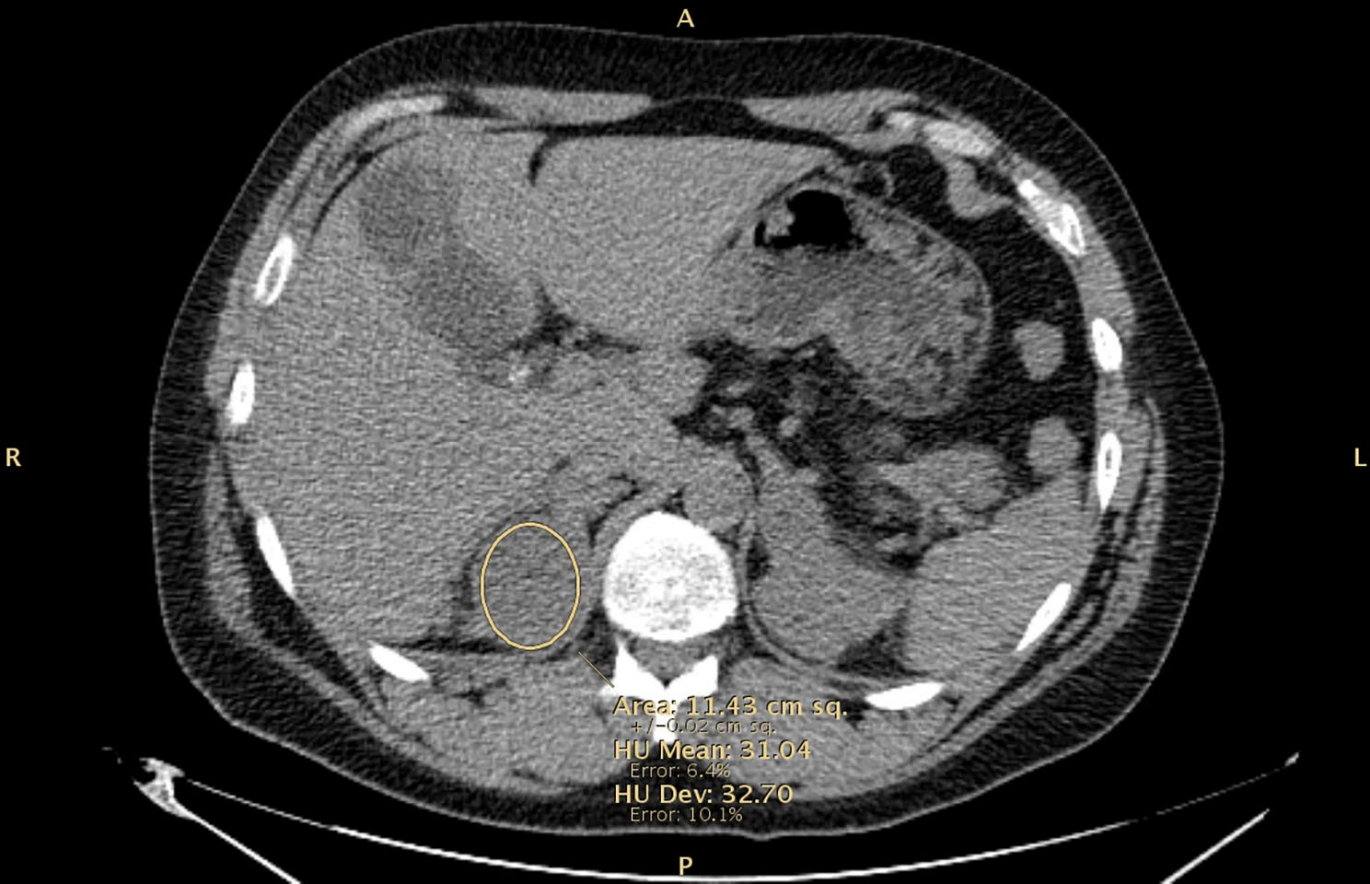
Bilateral adrenal masses at unenhanced CT scan with Hounsfield Unit (HU) of the right adrenal lesion

CT scans were discussed multidisciplinary; as in the right adrenal lesion Hounsfield units (HU) were elevated at unenhanced CT scan (31.04 HU) (Fig. 2) and a contrast uptake similar to the surgically removed sarcoma was detected, further investigations were performed in the suspicion of sarcoma metastasis.

**Fig. 3:**
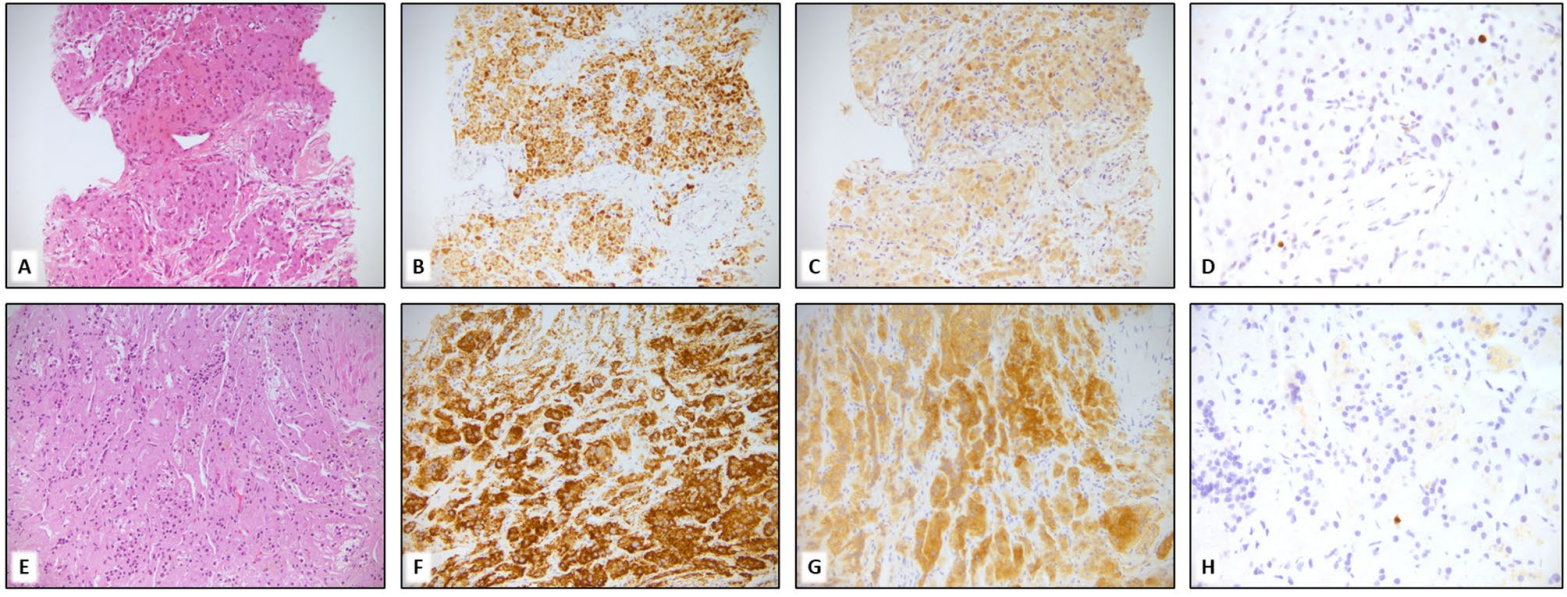
Biopsies of the peri-cecal mass (**A**) and adrenal lesion (**E**) show proliferation of cells with broad oncocytic and rarely clear cytoplasm, with bland cytology, lacking atypia and mitotic activity. Immuno histochemical staining for Melan A (**B**, **F**), Inhibin (**C**, **G**) confirm the corticosurrenal origin of the lesions. The proliferation index assessed with ki67 (**D**, **H**) is less than 1%. A, B,C, E,F, G magnification 200x. D, H magnification 400x

Abdominal positron emission tomography/magnetic resonance (PET/MR) imaging was performed, finding a great enhancement and uptake of the right adrenal gland; thus, a CT-guided biopsy of this lesion was performed to exclude the presence of sarcoma’s metastasis. The histology was consistent with adrenal hyperplasia (Fig. 3).

### Treatment

After our evaluation, the patient started glucocorticoid replacement therapy with dual-release hydrocortisone 20 mg once daily to improve disease control and avoid adrenal crisis. We decided on this option to increase treatment compliance since the patient reported overall well-being and was reluctant to take multiple drug doses.

### Outcome and follow-up

Dual-release hydrocortisone was well-tolerated, but hormone control remained poor; thus, we shifted to dexamethasone (0.37 mg/die) to obtain a greater suppression of adrenal androgen production and a potential shrinkage effect on the adrenal and ectopic lesions.

Therefore, we continued clinical and biochemical follow-up, noting a slight improvement of ACTH (51 ng/L) and 17-hydroxyprogesterone levels (1327 nmol/L) after one year; no adrenal crises were recorded during this period.

Moreover, the TART dimension remained stable at ultrasound testicular evaluation and the CT scan performed 12 months after the treatment shift showed the stability of adrenal and extra-adrenal lesions.

## Discussion and conclusions

This peculiar case report encompassed the wide spectrum of complications that occur in undertreated patients with classic CAH. Although CAH was diagnosed at birth and properly treated, the interruption of replacement therapy for almost thirty years led to chronic complications, as well as the risk of fatal adrenal crisis over those years. Surprisingly, the patient did not develop any adrenal crisis during this time frame nor during the surgery for sarcoma. This is probably due to the residual cortisol production and preserved mineralocorticoid pathway that is typical of SV CAH form; in fact, in SV there is a reduced risk of adrenal crises compared to the salt-wasting form [12].

The first interesting point in this case was the simultaneous discovery of a soft tissue sarcoma of the upper arm and bilateral adrenal enlargement in an unrecognized classic CAH; the history of a malignant tumor complicated the clinical and radiological evaluation since CT features of the adrenal lesions were highly suspicious.

Although adrenal enlargement due to excessive ACTH stimulation is frequent in patients with poor disease control, its malignant transformation has been rarely reported in CAH [13–19].

Indeed, bilateral adrenal lesions account for about 15% of adrenal incidentalomas and the main etiologies include adrenal metastasis from other tumors, primary bilateral macronodular adrenal hyperplasia and bilateral cortical adenomas, whilst CAH, pheochromocytomas, Cushing’s syndrome, primary malignancies, myelolipomas, infections or hemorrhage are less frequently encountered [20].

We speculated that the huge dimensions of the adrenal masses were related to the long discontinuation of medical treatment and consequent chronic stimulation mediated by high ACTH levels that, as previously reported, can also cause adrenal neoplasia, though rarely [13, 15–17]. Therefore, to completely exclude the possibility of malignant infiltration, we decided to perform an adrenal biopsy that confirmed the benignity of the lesions.

The second issue that makes this case report unique is that our patient is the second known case of CAH due to 21-OHD with adrenal rest tumors found in extra-gonadal site [11].

TART are quite usual complications in males with CAH, especially in patients with poor disease control, with an average prevalence of 40% [2]; in CAH patients, together with peripheral androgen aromatization and gonadotropin suppression, they are a major cause of infertility, causing obstructive.

azoospermia [1]. As previously told, ACTH is the major driver of TART growth but other factors seem to be involved in their formation, as a clear correlation with poor disease control has not always been proven [7].

There is a significant gender-related difference in the prevalence of adrenal remnants, with female patients presenting with OART only rarely [1, 21]; this is possibly related to the primary sex cord cells’ regression, during female embryogenesis, which includes also migrated adrenal cells [22].

However, while it is well recognized that adrenal rest tissue could develop in testicles and ovaries, a site different than gonads has only been described twice in literature, in a patient with CAH due to 3b-hydroxysteroid dehydrogenase deficiency revealing a large peri-renal adrenal rest tumor [10] and in a female with 21-OHD that harbored three nodules lying lateral to the fallopian tubes [11].

As known [23], adrenal glands develop near gonads in embryogenesis, thus it could be speculated that adrenocortical cells, which usually descend together with the testicles causing TART, could be found also in the retroperitoneal region, along the testicular descending pathway; that would explain why adrenal rests were discovered in our patient in peri-lumbar and peri-cecal sites.

In conclusion, impaired disease control in CAH induces significant bilateral adrenal enlargement that might pose significant clinical challenges during follow-up, especially in patients with a history of extra-adrenal malignancy.

Moreover, when CAH is not properly managed, in addition to TART and OART, an uncommon development of “ectopic” adrenal rest tumors in sites other than gonads might occur.

Indeed, we reported the first described case of a male with CAH due to 21-OHD with multiple unusual localization of adrenal rests.

This case report underlines the importance of adequate glucocorticoid replacement and life-long follow-up to prevent adrenal gland enlargements and adrenal rest tumors, which can interfere not only with fertility but also in the diagnostic workup of other concomitant clinical conditions.

## Data Availability

No datasets were generated or analysed during the current study.

## Abbreviations

21-OHD: 21-hydroxylase deficiency
ACTH: Adrenocorticotropic hormone
CAH: Congenital adrenal hyperplasia
CT: Computerized tomography
HU: Hounsfield unit
LH: Luteinizing hormone
MR: Magnetic resonance
OART: Ovarian adrenal rest tumor
PET/MR: Positron emission tomography/magnetic resonance
SV: Simple virilizing
TART: Testicular adrenal rest tumor
